# A comparative survey of the prevalence of human parasites found in fresh vegetables sold in supermarkets and open-aired markets in Accra, Ghana

**DOI:** 10.1186/1756-0500-7-836

**Published:** 2014-11-25

**Authors:** Kwabena O Duedu, Elizabeth A Yarnie, Patience B Tetteh-Quarcoo, Simon K Attah, Eric S Donkor, Patrick F Ayeh-Kumi

**Affiliations:** Department of Microbiology, University of Ghana Medical School, Korle-Bu, Accra, Ghana; Department of Medical Laboratory Sciences, School of Allied Health Sciences, University of Ghana, Accra, Ghana

**Keywords:** Contamination, Markets, Parasites, Vegetables

## Abstract

**Background:**

Consuming raw vegetables offers essential nutrients that one may not get when such vegetables are usually cooked. However, eating them raw may pose a great risk for transmissions of pathogens. Such risks may be influenced by the sources of the vegetables and washing techniques used. The aim of the study was to compare the prevalence and diversity of parasitic pathogens associated with vegetables sold at the two types of markets in Ghana and compare effectiveness of various washing techniques.

**Methods:**

We purchased two batches of samples of cabbage, sweet bell pepper, carrot, lettuce, tomato and onion within a two week interval. The vegetables were washed by three methods and the wash solution was concentrated and analyzed for parasites.

**Results:**

The prevalent parasites detected were *Strongyloides stercoralis* larvae (43%) and *Cryptosporidium parvum* oocyst (16%). Others present were Hookworm ova, *Entamoeba histolytica* cysts, *Giardia lamblia* cysts, *Cyclospora cayetanensis* oocysts, *Entamoeba coli* cysts, *Trichuris trichiuria* ova, *Enterobius vermicularis* ova, *Isospora belli* oocysts and *Fasciolopsis buski* ova. Contamination was highest in lettuce (61%) and cabbage and the least contaminated was tomato (18%). Contamination of vegetables sold at the open-aired markets was about ten-times that of the supermarkets.

**Conclusions:**

In Ghana, the large open-aired markets are the most patronized and serve as a supply point for most corner shops and stalls. The results thus highlight the potential of fresh vegetables serving as a major source of food-borne disease outbreaks and the contribution of open-aired markets to their transmission. Urgent public education on handling of fresh vegetables is recommended.

## Background

Parasitic infections lead to about 300 million severely ill individuals with approximately 200,000 deaths occurring in poor-resourced nations [1, 2]. Water and the food-chain have been reported as the main sources of outbreaks of diarrhea and other food-borne illnesses [3]. Though cooking at high temperatures are expected to kill most pathogens, this method may not apply to fresh vegetables which are rather eaten raw. Freshly eaten vegetables are a major source of nutrients like vitamins (vitamin B-complex, vitamin-C, vitamin A, and vitamin K) and minerals (calcium, magnesium, potassium, iron, beta-carotene) as well as dietary fiber. They protect the body against infectious diseases and serve as phytochemicals which function as antioxidants and anti-inflammatory agents reducing the risk of cardiovascular diseases, stroke and certain cancers [4, 5]. They also help prevent constipation, hemorrhoids, rectal fissures among others.

In Ghana, the food and water have been reported as the major routes of diarrhea outbreaks with vegetables being a major sources [6–9]. Of particular interest are intestinal protozoan infections like giardiasis, amoebiasis, cyclosporiasis and cryptosporidiosis which have caused high levels of morbidity and mortality [3, 9, 10]. Vegetables are sold mainly in open-aired markets though the influx of western culture and recent establishment of large supermarkets who sell vegetables have attracted some people. The cost of vegetables at supermarkets however cannot be compared to those sold in open aired markets which are cheaper and the average Ghanaian will patronize vegetables sold in open-aired markets rather than supermarkets.

Given the increased demand for ready-to-eat foods, particularly those containing uncooked fresh vegetables, there is great concern regarding the safety of these foods in the presence of unhygienic and improper management of the raw produce. Successful intervention strategies are therefore reliant on identifying not only the practices that are important for consumer protection, but also barriers that prevent consumers from responding to these interventions [11].

## Methods

### Study area and sampling

A total of 168 samples were collected in two rounds of purchases, two weeks apart. During each round, samples of carrot and onion (without leaves), fruits of tomato and green (bell) pepper and cabbages and lettuce were purchased from three supermarkets and three open-aired markets in the Accra metropolis. Supermarkets in Ghana have over the years been limited to the sale of factory-produced and packaged products. The open-aired markets were selected because they are noted for supplying not just to consumers but retailers as well. The three selected markets were designated X, Y and Z. Cleanliness of the vegetables was observed visually. Vegetables were ranked as unwashed (presence of dust or other particulate matter), washed (no visible form of dust) and washed and packaged.

### Sample preparation and detection of parasites

Samples were transported to the laboratory in sterile plastic bags. They were divided into three groups and each group washed with tap water, saline (0.85%) and phosphate buffered saline (PBS) [12]. About 100 g to 150 g of material was used for each vegetable except cabbage where one composite weighing about 400 g was used for each wash. In Ghana, vegetables are washed either with saline or tap water. Of the two, tap water is the most widely used and involves immersing vegetables in a bowl of tap water and cleaned several times with the hand or by vigorous agitation. Similarly samples were washed in the laboratory immersed in either physiological saline, solutions. About 500 ml of liquid was used to wash all vegetables except cabbages where 1000 ml of liquid was used to wash each composite. Lettuces were separated into individual leaves whereas cabbages were divided in four. The samples were agitated vigorously (manually) in the respective liquids for about three minutes. The washing solution was then transferred into sterile 50 mL conical tubes and centrifuged at 3000xg for 15 minutes. The supernatant was discarded and the deposit was examined.

Smears were made on grease-free microscope slides with the deposit from above. Six slides were made for each sample comprising two wet preparations each of unstained and Lugol’s iodine stained as well as two cold Ziehl Neelsen stained smears. In addition, the formol ether concentration technique [13] was also used for a portion of the sediments and slides prepared as indicated above. Slides were prepared and examined repeatedly until deposits were finished in the tubes.

### Data analysis and summary

Data was entered and cleaned in a Microsoft Excel database and appropriate descriptive statistics performed using GraphPad Prism (GraphPad Software, Inc, USA). One-way ANOVA was used to compare parasite distributions among all groups. Correlations were determined where appropriate. Statistical significance was set at *p-value <0.05*.

### Ethics

Ethical approval was obtained from the Ethics Committee of the School of Allied Health Sciences, University of Ghana. The sellers were not engaged in any forms of discussion. Data on how the vegetables are displayed for customers was obtained independently by observation by two researchers.

## Results

**Figure 1:**
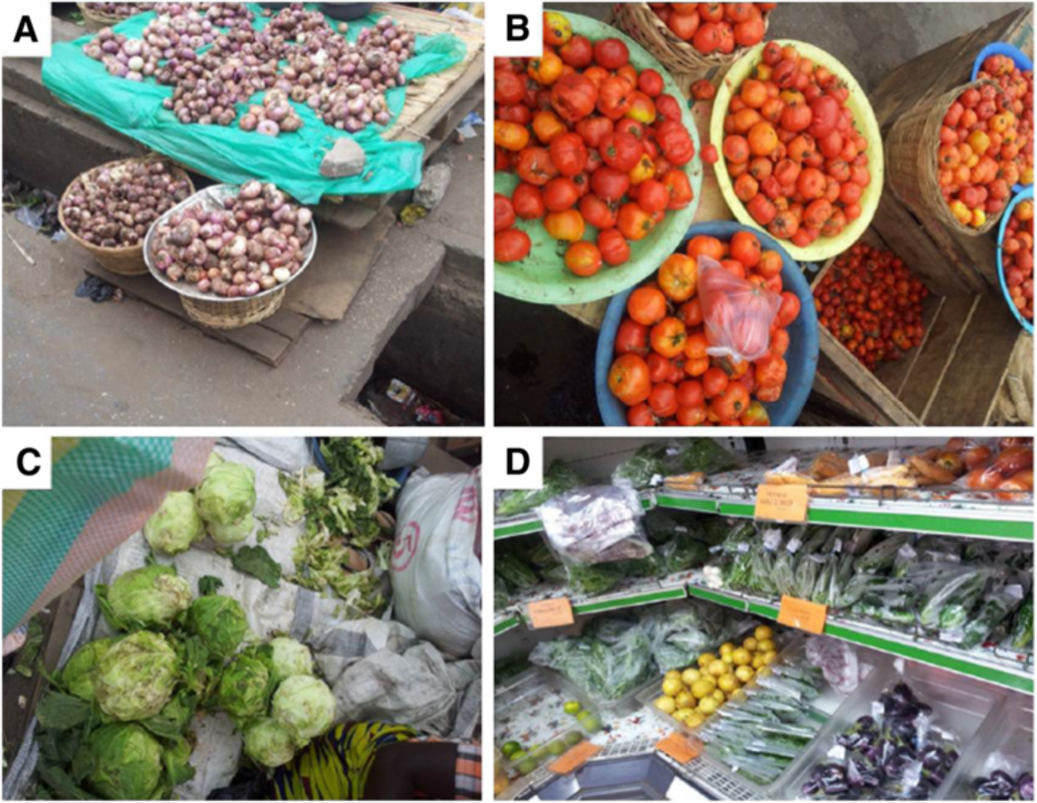
**Sample collection sites and forms of display.** Forms of which vegetables are displayed for customers to purchase. Onions **(A)**, Tomatoes **(B)** and Cabbage **(C)** as sold in the open markets. In the supermarkets **(D)**, vegetables are displayed and sold differently in visibly clean forms.

Samples from the open-aired markets were mainly kept in pans at display whereas those in the supermarkets were kept in an arranged shelve usually either packaged in plastic bags/containers or left on a stall (Figure 1). Samples from the supermarkets appeared ‘clean’. Samples from the open-aired markets although the sellers had washed some of them had some form of dirt on them. All sites were within central Accra.

**Figure 2:**
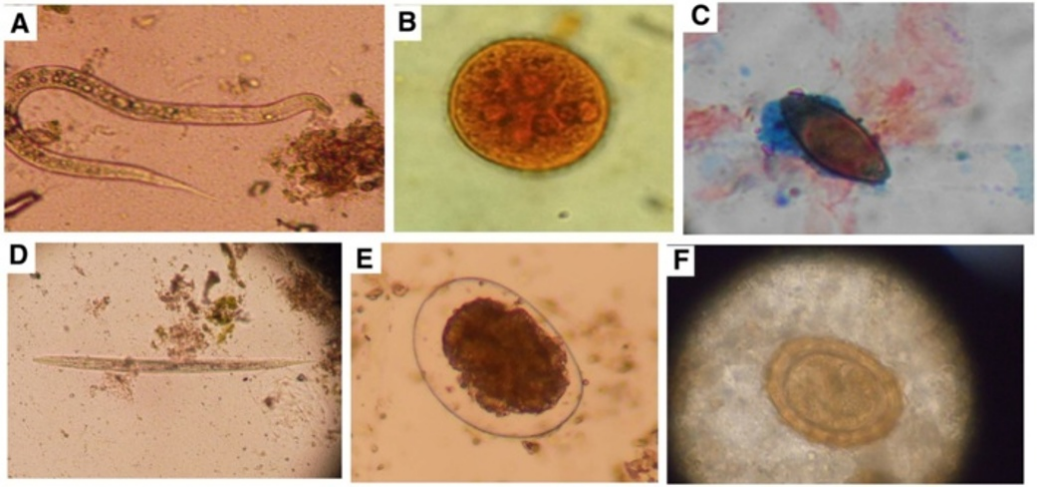
**Some of the parasites found in concentrated wet mounts of vegetable wash solutions in this survey. A**. Hookworm filariform larva; **B**. Entamoeba coli cyst; **C**. Trichuris trichiura ovum (on a ZN slide) **D**. Strongiloides stercoralis filariform larva **E**. Hookworm ovum; **F**. Fertilized ovum of Ascaris lumbricoides.

A total of six protozoans and five helminths were found to be associated with the various vegetables. This included *Cryptosporidium parvum, Entamoeba histolytica/dispar*, *Giardia lamblia*, *Cyclospora cayentenesis, Isospora belli, Entamoeba coli*, *Strongyloides stercoralis*, hookworm, *Trichuris trichiuria*, *Eterobious vermicularis*, and *Faciolopsis buski*. Some mites were also detected. Pictures of some of the parasites found in this survey is shown in Figure 2. Contamination ranged between 18% (tomatoes) and 61% (lettuces) with *C. parvum* and *S. stercoralis* being the most encountered protozoan and helminth respectively (Table 1). There was no significant difference between the means of the number of parasites detected in each group of vegetable although the variances differed significantly (p <0.0001). There was a significant difference between the mean (p =0.017) and variance (p <0.0001) of parasites encounter at the various markets. Very low prevalence of parasites was with vegetables obtained within the supermarkets and this was about ten times more among vegetables obtained from the open-aired markets (Table 1).

**Table 1:** **Abundance and prevalence of parasites associated with specific vegetables and study sites**

		Protozoa						Helminths							
	Vegetable	***Isospora beli***	***Cyclospora cayentanesis***oocyst	***Entamoeba coli***cyst	***Girdia lamblia***	***Entamoeba histolytica***	***Cryptosporidium parvum***oocyst	***Strongyloides stercoralis***	***Faciolopsis buski***	***Tricuris trihiuia***ova	Hookworm ova	***Enterobius vermicularis***ova	Total	Prevalence (%)	95% CI (%)
Cabbage		0	5	5	5	11	18	34	0	4	8	0	90	54	46 - 61
Green pepper		0	3	4	3	0	12	20	0	1	10	2	55	33	26 - 40
Carrot		0	3	0	4	4	6	21	0	2	4	3	47	28	22 - 35
Onion		0	3	2	3	2	9	35	0	0	15	1	70	42	34 - 49
Tomato		1	3	2	2	4	4	9	1	1	4	0	31	18	13 - 25
Lettuce		0	3	4	5	6	18	52	0	1	12	1	102	61	53 - 68
	Market														
OA Market	A	1	4	8	14	22	24	60	0	9	13	0	155	39	35 - 44
	B	0	10	9	8	0	20	40	0	0	24	6	117	30	25 - 34
	C	0	5	0	0	5	17	59	0	0	11	0	97	25	21 - 29
S’rmarket	X	0	0	0	0	0	2	4	1	0	0	1	8	2	1 - 4
	Y	0	1	0	0	0	3	4	0	0	2	0	10	3	1 - 5
	X	0	0	0	0	0	1	4	0	0	3	0	8	2	1 - 4
	Total	**1**	**20**	**17**	**22**	**27**	**67**	**171**	**1**	**9**	**53**	**7**	**395**		
	Prevalence (%)	0.3	5	4	6	7	17	43	0.3	2	13	2			

Of the three methods employed in washing vegetables, saline was the most effective and recovered 52% (95% CI = 47 - 57%) of parasites followed by PBS 34% (95% CI = 30 - 39%) and tap water 14% (95% CI = 11 - 18%). There was however some correlations between the methods used and the type of parasite recovered. The difference between the proportion of *E. histolytica, S. stercoralis, C. parvum and C. cayetanensis* recovered was statistically significant (p < 0.05). *Cryptosporidium parvum and E. histolytica* were the most parasites recovered in saline whereas *S. stercoralis* recovered most from PBS.

Overall, there was no correlation between the type of vegetable and the diversity of parasites found or the abundance of parasites. Neither was any statistically significant difference or correlation between the batches of vegetables bought from each site. However, storage conditions (refrigerated or not-refrigerated) as well as cleanliness of the vegetables at the time of purchase correlated with the diversity and abundance (p < 0.001 in all cases). Vendors from the open aired markets had washed tomatoes and lettuce. The vendors occasionally sprinkled water on the lettuce to maintain freshness. We however observed that the water was dirty. In addition to tomatoes and lettuce, vendors from markets B & C washed their carrots and pepper. There were no formal guidelines for these vendors to follow with respect to how clean they should present these vegetables although there appeared to be some consistency within each location. All vegetables from the supermarkets were washed, packaged in transparent plastic bags or containers and refrigerated. Vegetables from the open-aired markets had the highest diversity of parasites (Figure 2). The diversity was however not the same at the sites during consecutive visits and sampling.

## Discussion

Results from this study shows that, the prevalence of protozoan and helminth parasites in the fresh vegetable chain in Accra is high. This finding is particularly worrying as the problem lingers on. Similar reports have made in some parts of Nigeria [14] and Ghana [15, 16]. Notably, this study has seen a higher prevalence for *S. stercoralis* than *C. parvum. Cryptosporidium* associated diarrhea has been reported frequently among various groups of Ghanaians [17, 18]. Despite these reports, the problem appears not to have improved in recent times. Another reoccurrence is the high prevalence of parasites associated with lettuce [19, 20]. There are reports of a wide variation of parasites associated with leafy vegetables (cabbage and lettuce) than the other vegetables which may be due to exposure of the leaves to the soil surface or the nature and appearance of the vegetable [21]. The high prevalence of *C. parvum* could be associated with contaminated water used for irrigation while *S. stercoralis* and hookworm can be associated with contaminated human excreta used as manure [22]. High contamination of lettuce maybe due to how the vegetable grows on the farms, precisely on the surface of the soil unprotected, due to the curly nature of the leaves. This makes it possible for them to be associated with most parasites especially geohelminths such as *S. stercoralis*, hookworm and *T. trichiuria* which are soil transmitted nematodes [23]. Also the softness and fragility of the leaves of the lettuce makes most vendors ignore washing it rigorously in order to maintain the freshness of their lettuce thus making them a potential source of parasitic infections.

We found infective stages of some of the parasites in this survey (eg. *S. stercoralis* filariform larva, cysts of *E. histolytica/dispar* and *E. coli*, *G. lamblia* cysts, etc.). The presence of infective stages poses a greater health risk from handling and consuming the contaminated vegetables. *S. stercoralis* filariform larva could penetrate the skin of both the vendors as well as consumers. Although non-infective stages where also found for some parasites (e.g. *T. trichiura* unembryonated ovum, unembryonated *Ascaris* ovum, etc.), these life forms could potentially develop into infective stages over time and pose significant health risks.

Though vendors may get their supplies from the same source, our study shows that, parasitic contamination may not involve the same diversity of parasites for each supply. Particularly, we noticed increases in the diversity of parasites during the second visit to the open aired markets. The differences were also seen for the relative abundances of the parasites found in the vegetables. These differences may be due to differences in the way these vegetables are handled from the farms to the market place. Although wastewater re-use for peri-urban agriculture is encouraged for economic purposes [22, 24, 25], significant infection associated dangers are reported [26]. With the lack of or inadequate treatment systems, wastewater use for agriculture presents a huge public health risk in most developing and underdeveloped countries. We have also shown that, washing vegetables (or fruits) with just water is not enough to remove any contaminating parasites. It is common to find many people washing fruits and vegetables with just water prior to eating. This practice could potentially lead to inadequate washing and thereby cause infection from any contaminating pathogen. The need for washing vegetables and fruits with saline is strongly encouraged.

## Conclusions

This study highlights that, the potential of fresh vegetables serving as sources of infection with various pathogens is still high. Washing of vegetables with just water is inadequate to remove all contaminating pathogens. Vegetables obtained from open-aired markets are the most contaminated compared to those from supermarkets, hence, both vendors and consumers should be careful when handling them to avoid infection. There’s urgent need for public education on safe and proper handling of fresh vegetables.

### Limitations

Identification of *C. parvum* is rather difficult. In this study, we relied solely on the modified ZN staining and the experience of the microscopists. This is a key limitation as yeasts in water may stain similarly to the oocysts. Although this study presents some important public health concerns related to food safety, the relatively small sample size is a limitation. The need for further studies using a larger sample size from more diverse sampling sites is strongly encouraged.

## Authors information

Present address of KOD is the Institute of Cell Biology, School of Biological Sciences, University of Edinburgh, UK.
